# iEngage: A digital health education program designed to enhance physical activity in young adolescents

**DOI:** 10.1371/journal.pone.0274644

**Published:** 2022-10-05

**Authors:** Corinne Caillaud, Susan Ledger, Claudio Diaz, Gaël Clerc, Olivier Galy, Kalina Yacef

**Affiliations:** 1 Discipline of Biomedical Informatics and Digital Health, School of Medical Sciences, Faculty of Medicine and Health, The University of Sydney, Sydney, NSW, Australia; 2 Charles Perkins Centre, The University of Sydney, Sydney, NSW, Australia; 3 Department of Respiratory and Sleep Medicine, Centre for Sleep Health & Research, Royal North Shore Hospital, St Leonards, NSW, Australia; 4 School of Computer Science, Faculty of Engineering, The University of Sydney, Sydney, NSW, Australia; 5 Bepatient Australia Pty. Ltd. (Alira Health), Rozelle, NSW, Australia; 6 Interdisciplinary Laboratory for Research in Education, EA 7483, School of Education, University of New Caledonia, Nouméa, New Caledonia; Universiti Malaya, MALAYSIA

## Abstract

iEngage is a modular health education and behavioural change program designed to help adolescents increase moderate to vigorous physical activity (MVPA). The program is delivered through the iEngage app which integrates activity trackers data (Misfit Ray^©^) within 10 interactive learning modules. Key features include guidance to set goals, self-monitor and assess achievements, and experiential learning via the connected activity trackers which allows for continuous steps recording during the program. iEngage was implemented in two schools over 5 weeks with 10–12 years old adolescents (n = 57) and PA outcomes compared to control group (n = 26). Results show that adolescents successfully set goals and self-assessed achievements during the program, progressing toward higher physical activity (PA) levels as shown by the 30% increase in daily steps through the program (+ 2647 steps/day, P < .001) with boys increasing goals and achievements faster than girls. The consistency in days totalling at least 11,000 steps/day increased from 35% at the start to 48% at the end of the program. The increase in PA is confirmed through the assessment of MVPA during schooldays pre- and post- program via research grade wrist accelerometers in both iEngage and control participants. Contrasting with the control group, MVPA was increased in the week following the program (~+5 min/day, P = .023) in short bouts, particularly during lunch time, recess and after school. This study shows that a digital program integrating activity trackers data, health education, goals setting and self-monitoring of PA, helped young adolescents enhance PA goals, improve achievements and increase MVPA.

## Introduction

Physical activity (PA) is a major component of health and wellbeing across the lifespan and contributes to reducing all-cause mortality. However, current evidence from large population cross-sectional surveys indicates that 81% of children and adolescents globally do not participate in sufficient amounts of PA [1]. Australian guidelines (Australian Government, Department of Health) recommend that children and adolescents perform at least 60 minutes of moderate to vigorous PA (MVPA) each day [2]. Surveys showed that only 20% of 5–17 years-old Australians meet these recommendations, with no improvement over the past 10 years despite a number of public health campaigns and interventions [3]. Pooled accelerometry data from several countries including Australia showed that adolescents accumulated on average 30–35 min of MVPA daily with only ~ 7% of 9–13 years meeting the WHO recommendations for PA [4].

Because low participation in PA is a risk factor for the development of overweight and obesity in young people, leading to adverse cardiometabolic profiles, there is an urgent need to identify approaches and interventions that are effective [5, 6]. For instance, a large study including ~21,000 children and adolescents found that MVPA was significantly and inversely associated with cardiometabolic outcomes independently of time spent sedentary, demonstrating the importance of focusing on increasing MVPA [7]. Studies also show that PA behaviour carries on from childhood to adulthood, with insufficient PA levels during adolescence leading to low PA levels in adulthood [8, 9], indicating that active behaviours must be adopted as early as possible to promote a healthy lifestyle across the whole lifespan. Importantly, the overall low level of participation in MVPA is partly driven by perceived lack of support, poor motivation, low physical competence [10], poor health knowledge or limited ability to organise own PA [11].

Numerous interventions have attempted to enhance participation in PA and MVPA in children and adolescents; most of them showing no or small changes in PA behaviour in response to interventions, indicating that changing PA behaviour in adolescents is challenging [12]. School-based PA interventions may be more effective at increasing the proportion of adolescents who engage in MVPA, however adherence and magnitude of change are generally low [13]. The rise of technology and the availability of accelerometers are real opportunities to facilitate objective self-assessment of PA providing relevant information to support the adolescents’ engagement with PA interventions [14]. Regrettably, the overall moderate quality of existing commercial health and fitness apps, the poor app usage as well as the inconsistent use of behavioural change techniques (BCTs) have been identified as barriers to the efficacy of app-based intervention in children and adolescents [15]. For example, existing apps do not take advantage of the technology to continuously collect PA data during the program or to use these PA data as a mechanism to provide feedback to adolescents during the program [15]. It has been recommended that future PA apps must be specifically designed for the target population, combine BCTs (such as goal setting, self-monitoring and performance feedback), provide health education and behavioural change advice, and incorporate some gamification [15]. With the aim to tackle this gap, we designed a health education program delivered through a digital app, integrated with activity sensors.

In this article, we present our approach to designing and implementing iEngage, an educational and behavioural change program tailored to 10–12 years old adolescents. Building on an initial work [17], the program combines experiential learning and BCTs and is delivered via a user-friendly digital app connected to activity trackers.

We hypothesised that iEngage, combining experiential learning and behavioural change techniques, delivered via a user-friendly digital environment with integrated activity trackers, will be able to help adolescents enhance PA behaviour.

We present the original design approach of the iEngage program and report on its effectiveness for enhancing PA goals and achievements and increase MVPA in daily life of 10–12-year-old Australian adolescents.

## Method

### iEngage: Approach and framework

iEngage is an evidence-based health education and behavioural change program designed to help young adolescents increase MVPA. The program is supported by a digital environment that includes the iEngage app connected to activity trackers. Using live data from these trackers and digital health education modules, iEngage aims to drive behavioural change through experiential learning, goal setting and self-assessment of achievements. The program and companion app were co-designed with experts in physical education, exercise science, data science and mobile health industry experts. A few short sessions were organised to gather feedback from adolescents.

**Fig 1:**
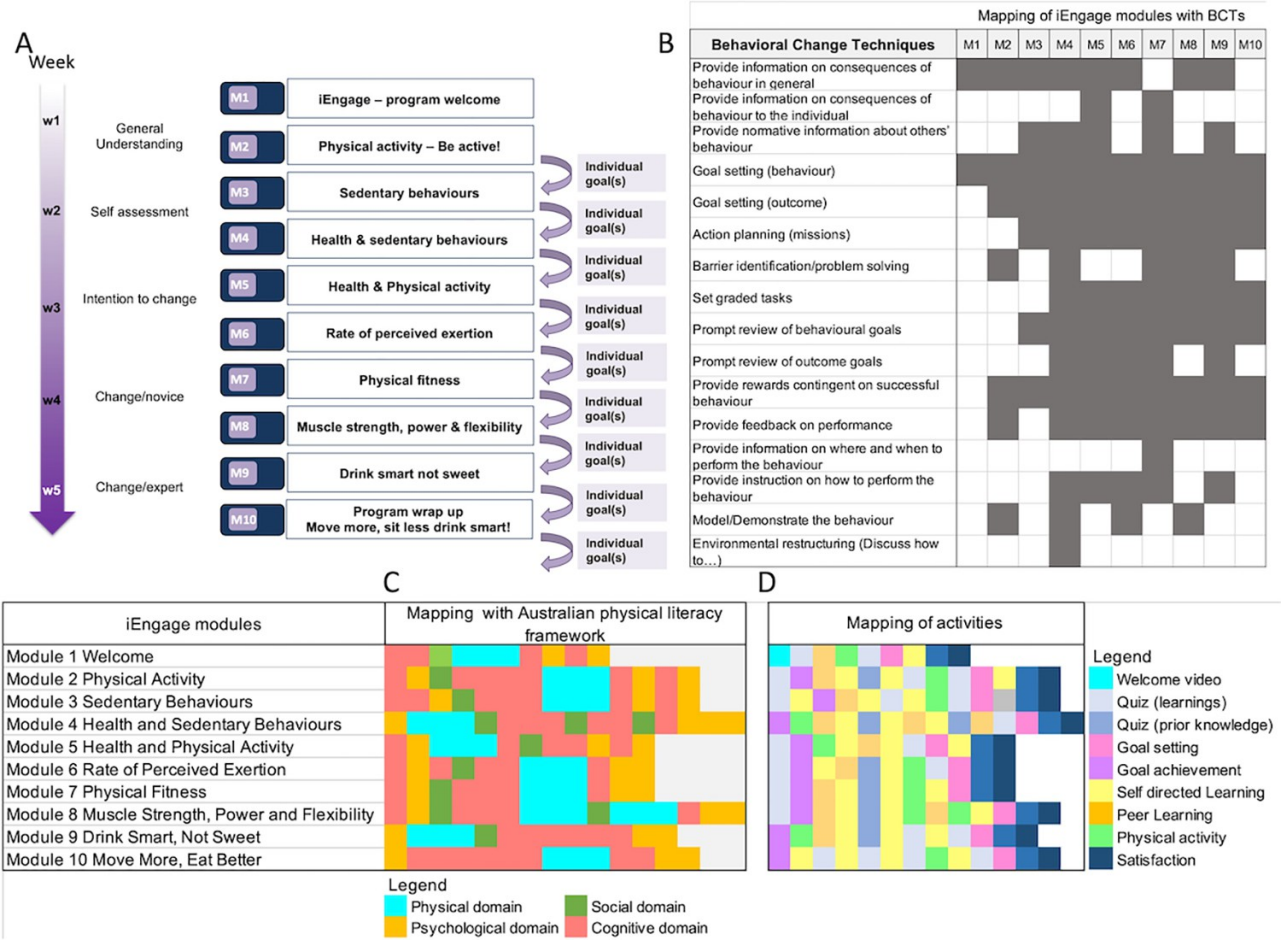
iEngage structure. Description of the content of each module and the progression through the various stages of behaviour change. Panel A: Individual goal setting (daily steps goals and missions) started in module 2. Panel B: Mapping of each module content with behavioural change techniques. Panel C: mapping with the Australian physical literacy framework. Each square represents an activity which is colour coded to show mapping with the framework. Panel D: mapping of all activities within each module across the program. Each activity is coded as per the legend.

Topics such as health, intensity of PA, MVPA, sedentary behaviours (SB), physical fitness, physical effort, exertion, mode of exercise or the impact of PA on body functions are explained through a series of 10 digital modules (Fig 1A) while activity trackers help connecting concepts with experience. iEngage is grounded in evidence-based principles and practices in education and physical education and builds on BCTs. We used a refined taxonomy of BCTs, the “Coventry, Aberdeen & London—Refined” (CALO-RE) taxonomy, which is specifically adapted to help people change their PA and health behaviours [16]. The CALO-RE taxonomy comprises 40 items amongst which we identified 16 items relevant to our objectives, the population targeted and the school setting in which we deployed the program, based on current evidence that schools provide a good setting for PA interventions [13]. The 16 items were mapped to each module of the iEngage program (Fig 1B and 1C).

iEngage also aligns with the Australian Physical Literacy Framework. Physical literacy is described as the skills, knowledge and behaviours that help us lead active lives [2, 3, 17, 18]. It integrates physical, psychological, social and cognitive domains that can be developed holistically across the lifespan from pre-foundational to mastery level. These domains are embedded in each iEngage module (Fig 1C). In addition, through an experiential learning approach, iEngage supports skill building in recognising and setting internal standards of PA intensities. This is done through understanding how intensity of actual PA relates to perceived exertion. As part of each module, participants practice short bouts (5 to 10 min) of light, moderate and vigorous PA to build practical knowledge and gain experience of diverse PA intensities. Throughout the program, participants learn to assess exercise intensity using perceived rate of exertion and comparing their ratings with the information provided in the app via activity trackers. Each module involves BCTs as well as learning activities such as quizzes, self-assessment of goals’ achievement, individual learning, peer-learning, and goal setting (Fig 1B and 1D). The general organisation of each module follows a consistent structure to facilitate adolescents’ engagement with the app. Consistency across modules reduces the cognitive effort in understanding the tasks, thus helps with engagement [19].

### Goal setting

The process for personalised goal setting and assessment of achievements is designed as follows: in each module, the adolescent must set two types of goals for the next 3 days through two tasks: (1) choosing the *amount of daily steps and MVPA* they aim for (quantitative goal) and (2) choosing a *mission* from four suggestions (qualitative goal). To facilitate the quantitative goal, the app proposes a finite list of increasing commitments to choose from, with increasing boundaries throughout the program. Step choices from modules 1 to 10 are: 5,000 to 7,000 steps in module 1; 6,000 to 10,000 steps in module 2 and so on. MVPA choices start from module 6 and increase from 20 min to 60-min daily of MVPA as follows: 9000 steps/day and 20 min of MVPA, 10000 steps/day and 30 min of MVPA, 11,000 steps/day and 40 min of MVPA, 12000 steps/day and 60 min of MVPA. The maximum number of steps is based on studies estimating that performing 12,000 steps/day is possible only if adolescents engage in at least 45 minutes of MVPA [20].

The missions encourage adolescents to engage with the program through diverse activities with their parents or siblings, as research shows the importance of family, friends and community in driving behaviour change in adolescents [21]. Examples of missions include sharing with family members their learning from the program, playing a sport game with friends or siblings, spending less time in sedentary activities, participating in more active play during recess at school or identifying small changes in their environment that could help them be more active.

### Self-assessment of achievements against goals

In each module but the first one, the adolescent assesses their achievements against their individual goals. This involves: 1) reading the number of steps recorded daily via the activity trackers (Misfit Ray^(C)^) in the iEngage app; 2) reporting achievements in the app, 3) providing a self-assessment of achievements by answering a quiz and 4) reporting on completion of their mission. It is made clear in module 1 that iEngage focuses on progressing and achieving individual goals. iEngage has no component involving ranking the adolescents’ achievements and it has no competitive aspect. This is aligned with studies showing that competition and performance-based ranking can lead to adverse outcomes in adolescents [22].

### Learning activities

**Fig 2:**
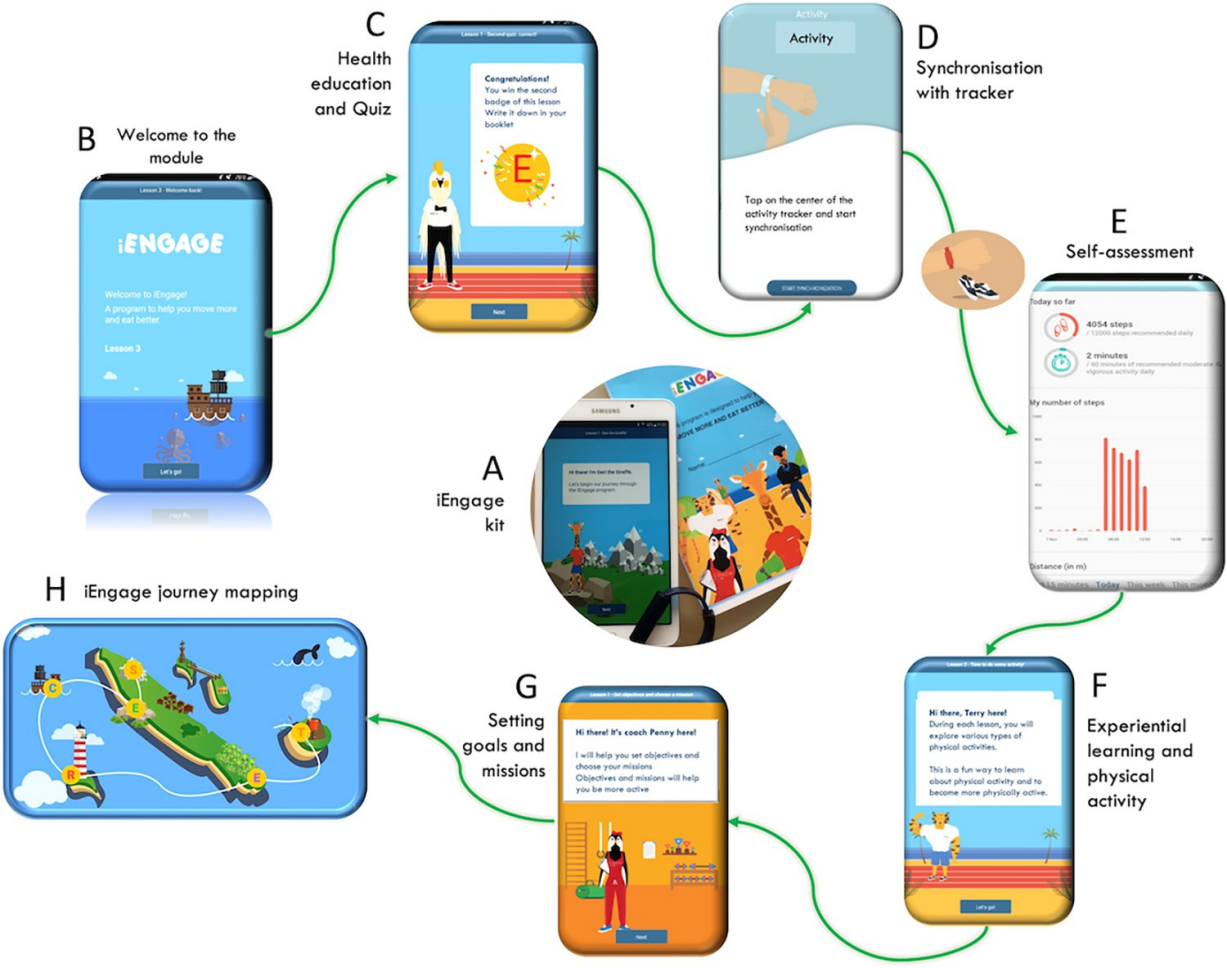
iEngage design. Design of the iEngage app interface and overview of the user experience. The iEngage kit including a tablet, a Misfit Ray activity tracker, earphones and a booklet (A). The digital environment supporting the diverse activities (B to H) included a welcome screen displaying learning objectives (B), health education, quiz and badges reward (C), synchronisation with trackers (D), self-assessment and goals setting (E) that included visuals of daily steps and time spent in MVPA, experiential learning (F), goals setting and mission (G) and the iEngage journey mapping showing progress toward the iEngage secret message (H).

Knowledge acquisition involves either quiet individual self-paced learning activities such as reading, watching videos, and answering quizzes, or peer-learning through open questions that adolescents discuss with their friends in the classroom or outdoor while walking in the playground. Each module includes some physical exercises, with sessions lasting 5- to 15-min. Physical activity is proposed as an experiential learning activity and skill acquisition, not as an exercise training session. The maximal amount of additional PA due to the program’s activities during a module is 15 minutes [23]. The school did not propose additional physical education session during the program. iEngage embeds gamification features as a strategy to enhance engagement and motivation: achieving goals, answering correctly to quizzes, or participation in the physical exercises, all provide opportunities to win points that unlock a badge. Each badge discloses a letter that will make a word by the end of the module and a full sentence (the iEngage moto) by the end of the program. While this strategy aims at promoting engagement with each module through the recognition of achievements across knowledge and behaviours, it is also used to develop a narrative across the program. Indeed, each completed module progresses the adolescent in their iEngage journey which is mapped as an expedition around an imaginary island. A map showing their progress is presented at the end of each module (Fig 2F).

### iEngage app: Design and mode of delivery

A key feature of the iEngage program lies in the digital integration via the iEngage app and activity trackers worn throughout the program, providing 5 weeks of continuous PA data. The app interface is specifically designed to promote positive and inclusive user experience for 9–12 years old adolescents and develops a narrative on PA and health through a creative and colourful design. It brings into play five animal mascots, each dedicated to a specific activity (knowledge sharing, tips and skills learning, quiz, tracker synchronisation, goal setting and choosing missions, experiential activity). They signal the start of a specific activity, thus helping the transition between activities (Fig 2B to 2H). An animated video embedded in the first module explains the program. The iEngage kit is made of a digital tablet, a booklet where adolescents record information manually in writing during each module and add summary stickers, a Misfit Ray^©^ activity tracker and earphones (Fig 2A).

### Delivering the iEngage program: Study design

The research protocol and consent procedures were approved by The University of Sydney Human Research Ethics Committee (2017/272) and by the NSW Department of Education State Education Research Applications Process (2017151).

The study was conducted in 2017 and 2018 with a sample of one control class (26 students) and 2 intervention classes (57 students) in schools located in the Sydney inner city area. To avoid information contamination, the study groups were conducted in two different schools and years. Written consent was obtained from the principals, class teachers, parents and students. All participants were consenting students attending a Year 5/6 class and were able to withdraw from the study at any time. The 2017 and 2018 studies were conducted over the same month (November) of each year and the data were combined for analysis. The program was delivered over 5 consecutive weeks. The research team attended the school to supervise the delivery of the program in the morning, usually between 9AM and 11AM. The control class received no program over the 5 weeks.

The participants’ MVPA was objectively measured prior the program and after completion of the last iEngage module using a research grade accelerometer validated in young adolescents (GENEActiv) [24]. The GENEActiv accelerometer was worn on non-dominant wrist for three complete school days and nights. Data was collected at 60 Hz, downloaded using the GENEActiv software (Version 3.2) as 1-sec Epoch and analysed using 3-sec bouts [25]. The participants also completed a self-report questionnaire on their physical activity over the previous 7 days (C-PAQ) [26], providing a subjective measure of their physical activity. Baseline measurements including anthropometry (age, height, weight, waist circumference) and physical fitness tests (20m multistage shuttle test, flexibility, hand grip, vertical jump, agility) were performed two weeks prior to starting the program in accordance with existing standards as previously described [27].

For the iEngage classes participants wore a Misfit Ray^©^ activity tracker continuously for the 5-week duration of the program on the non-dominant wrist. Misfit Ray^©^ is a commercial tracker well tolerated by children and adolescents. Daily step counts were provided to the participant via the iEngage app during each module. In addition, raw data, averaged over 1 min periods were downloaded at the end of the program to allow analysis of daily steps consistency rate as previously described [23]. Goals, self-assessment, answers to quizzes, data from the activity tracker, and free text were all recorded in the iEngage app. The data from each module provided information on students’ engagement with the program through goal setting and self-assessment of achievements. Self-reported data are reported as the percentage of participants achieving either personal goals, or the 11,000-step/day target.

### Statistical analysis

Data analysis are presented for twenty-six control participants and fifty-seven iEngage participants. Seventy-one participants were included in the iEngage group. Fourteen were excluded from the analysis because they missed at least 3 modules due to absences, injury, or attendance to special study programs. No student dropped-out or asked to stop the program. Anthropometric, fitness and PA data from the activity tracker (daily steps collected at each module for 5 weeks) as well as GENEActiv data (collected and averaged over 3 school days before and after the iEngage program) are presented as mean +/- standard deviation (SD). Goals and achievements are presented as a percentage of students choosing or achieving a particular goal or PA level. To assess goal achievement for the whole cohort, data was aggregated over the 3 days preceding each module (57 participants x 3 days, Fig 4). Missing data from the activity tracker dataset was imputed using regression analysis. Differences between groups and gender with regards to anthropometry and physical fitness were analysed using a two-way ANOVA (group x gender). Goals and achievements data were analysed using a Pearson Chi-square statistical test for goodness-of-fit. A one-way ANOVA (gender) with repeated measures was used to test goals progression during the program. Pre- post- MVPA was analysed in 23 control and 41 iEngage participants GENEActiv datasets (only full dataset were used in the analysis, i.e. three full days of data both pre and post program). A two-way ANOVA with repeated measures (gender x group) was used to test the impact of iEngage on PA as assessed by GENEActiv sensor before and after the iEngage program. The statistical analysis was conducted using IBM SPSS Statistics 24 Software (New York, USA). Statistical significance was set at alpha level = 0.05.

## Results

### Descriptive and baseline data

There was no statistical difference between control and experimental groups at baseline for anthropometric data, physical activity or physical fitness (Table 1). Most participants (77%) achieved aerobic fitness meeting the Health Fitness Zone criteria [28]. Self-reported PA (PA Questionnaire) showed higher PA levels in boys and no difference between groups. Objective PA assessment via GENEActiv accelerometers showed that average daily MVPA for all participants at baseline was 50 ± 21.3 min/day with only 32% of participants achieving 60-min /day of MVPA. Overall, boys spent more time in MVPA at baseline (62.8 ± 23.2 min/day) than girls (43.3 ± 17.7 min/day) (F(1, 5862) = 14.7, *P* < .001).

**Table 1:** Participants’ age, physical measures and physical fitness (G: Girls, B: Boys).

Group	n	Age	Weight	Height	Waist ^$^	% fat	BMI	waist to height ^$^	VO_2_max (ml/kg/min)	Running Speed (km/h)	Agility	Vertical Jump	Flexibility ^$^	Hand Grip ^$^
		(y)	(kg)	(cm)	(cm)		(kg/m^2^)				(s)	(cm)	(cm)	(kg)
**Control**														
**G**	16	**10.4**	**37.4**	**144.5**	**66.6**	**21.8**	**17.8**	**0.46**	**46.4**	**10.2**	**14.6**	**25.9**	**23.0**	**14.3**
		0.5	7.9	6.9	7.1	5.1	2.5	0.03	5.4	1.0	1.1	5.6	8.1	4.3
**B**	10	**10.4**	**45.2**	**147.5**	**77.6**	**24.4**	**20.7**	**0.53**	**45.5**	**9.9**	**14.3**	**26.7**	**10.1**	**13.8**
		0.5	12.5	5.2	11.9	8.3	5.3	0.08	4.0	0.9	1.9	6.2	9.3	5.1
**ALL**	26	**10.4**	**40.4**	**145.6**	**70.8**	**22.8**	**18.9**	**0.49**	**46.1**	**10.1**	**14.5**	**26.2**	**18.0**	**14.1**
		0.5	10.4	6.4	10.6	6.5	4.0	0.06	4.8	1.0	1.3	5.7	10.6	4.5
**iEngage intervention**														
**G**	29	**11.2**	**44.2**	**150.7**	**69.2**	**23.6**	**19.3**	**0.46**	**45.7**	**10.3**	**13.9**	**27.6**	**25.8**	**21.2**
		0.6	11.4	7.7	9.7	6.9	3.5	0.05	4.7	0.9	0.9	6.2	8.4	4.7
**B**	28	**10.7**	**42.7**	**147.8**	**71.2**	**20.5**	**19.2**	**0.48**	**44.2**	**9.8**	**14.1**	**27.3**	**15.3**	**17.3**
		0.7	11.8	7.6	10.5	8.4	3.7	0.06	5.3	1.0	1.7	8.7	6.1	4.7
**ALL**	57	**10.9** *	**43.5**	**149.3**	**70.5**	**22.1**	**19.2**	**0.47**	**45.0**	**10.1**	**14.0**	**27.4**	**20.6**	**19.3**
		0.7	11.5	7.7	10.0	7.8	3.7	0.06	5.0	1.0	1.4	7.5	9.0	5.1

*: Control group compared to iEngage group (age: *P* < .001, hand grip: *P* < .001)

$: Girls compared to boys (waist: *P* = .041; waist to height: *P* = .007: Flexibility: P < .001)

### Analysis of participants physical activity goals and self-assessment of achievements

**Fig 3:**
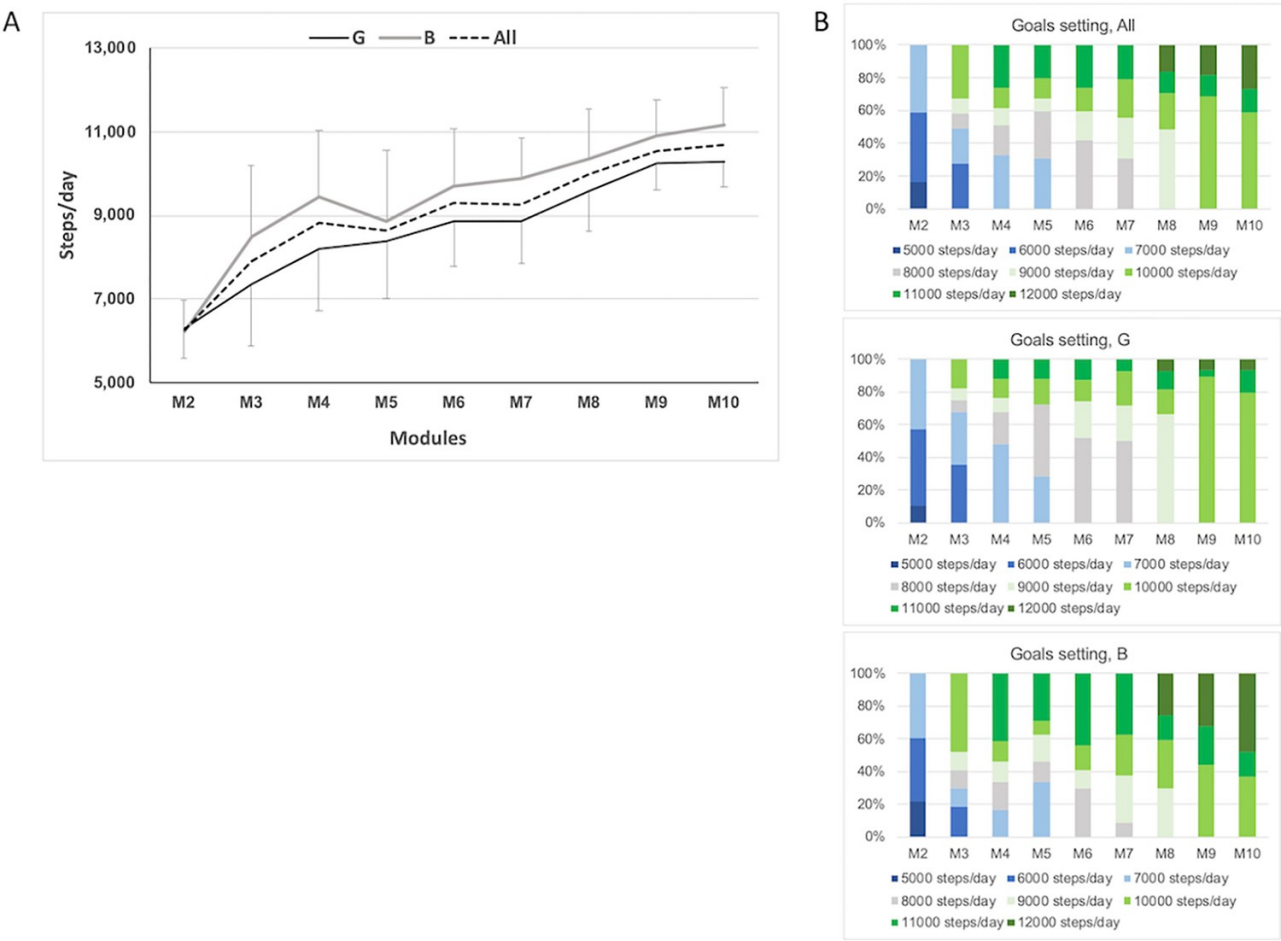
Goals setting. Panel A: average daily steps goals set by adolescents over the course of the program for each module. Goals increased from module 2 to module 10 (Wilks’ Lambda F(8,17) = 91.1, P<0.001). There was a significant gender effect (F(1, 24) = 4.46, P<0.05) with boys aiming for higher daily steps goals. Panel B: proportion of adolescents choosing a given step goal for each module. Note: From M6, goals also included a minimum amount of MVPA (10,000 steps and at least 15 min MVPA, 11,000 steps and at least 15 min MVPA, 12,000 steps and at least 30 min MVPA). All: all participants (n = 57), B: boys (n = 28), G: girls (n = 29).

On average, participants progressively and significantly increased their daily steps goals from 6,153 ± 774 steps/day in M2 to 9,153 ± 1317 in M6 and finally 10,461 ± 760 in M10 (Wilks’ Lambda F(8,17) = 91.1, *P* < .001, Fig 3A). We found that 9% of participants set their goals at 9,000 steps/day in M3 while 48% did so in M8 showing progression in PA goals (Fig 3B).

While participants could select 12,000 steps/day from module 8, 17% of participants set this goal in M8 compared to 27% in M10, with a marked difference between girls (7%) and boys (48%) (Fig 3B). Across the program, boys set goals higher than girls by about 800 steps, aiming for ~11,200 steps at the end of the iEngage program (F(1, 24) = 4.46, *P* = 0.045, Fig 3A and 3B).

**Fig 4:**
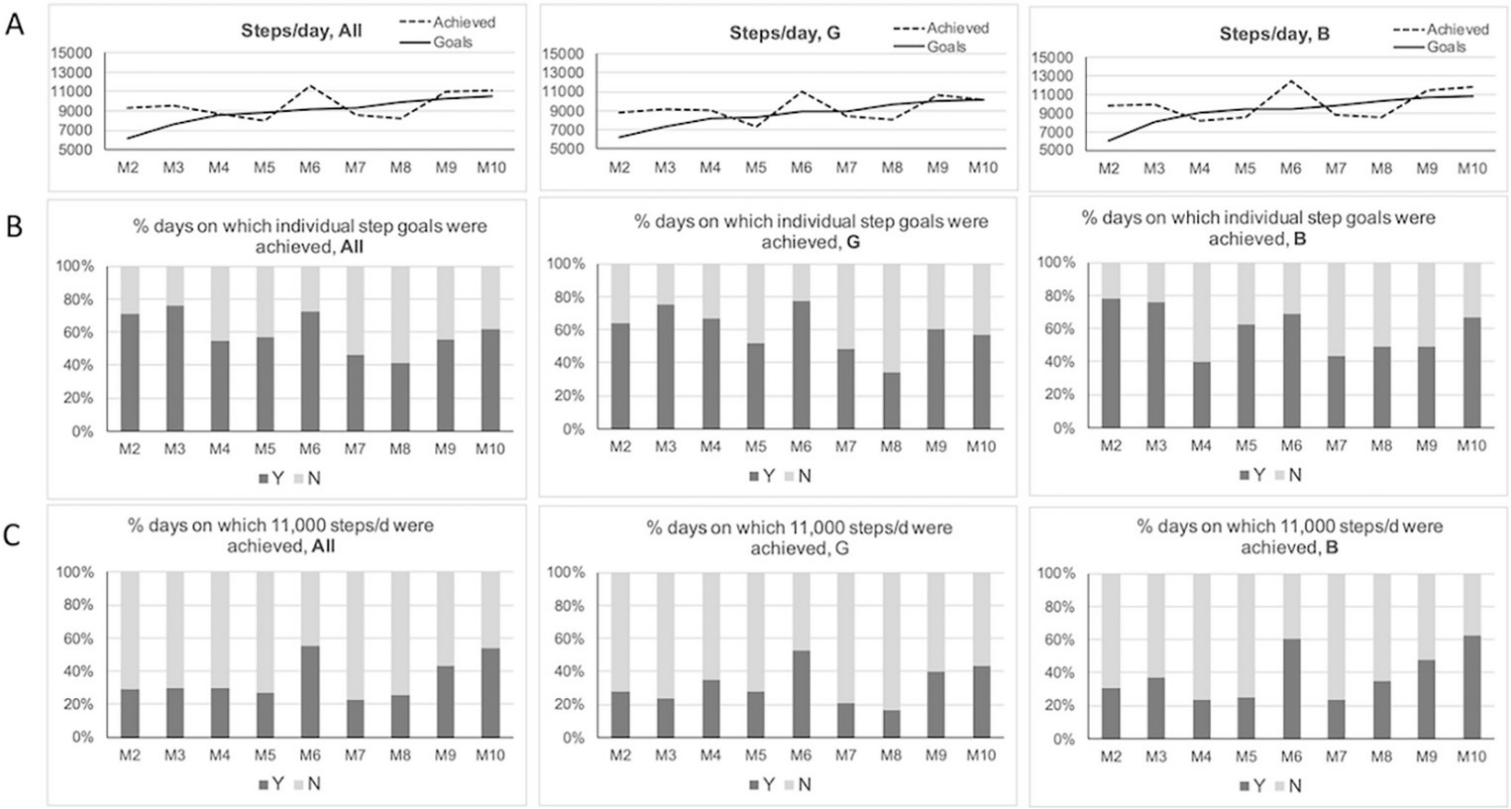
Self-reported achievements. Daily steps reported by the adolescents in each module. The step value reported for each module is the average of daily steps performed during the 2 or 3 previous days (between 2 modules). Panel A: averaged daily steps goals and achievements reported by adolescents in the app as part of each module’s self-monitoring activities. Panel B: proportion of days on which individual step goals were achieved (based on self-report of step data). Panel C: % of days on which 11,000 steps/d were achieved calculated from self-reported step data. **: P<0.01, Chi Square test. Compared to predicted form M2 (30% Yes and 70% No). All: all participants (n = 57), B: boys (n = 28), G: girls (n = 29).

**Fig 5:**
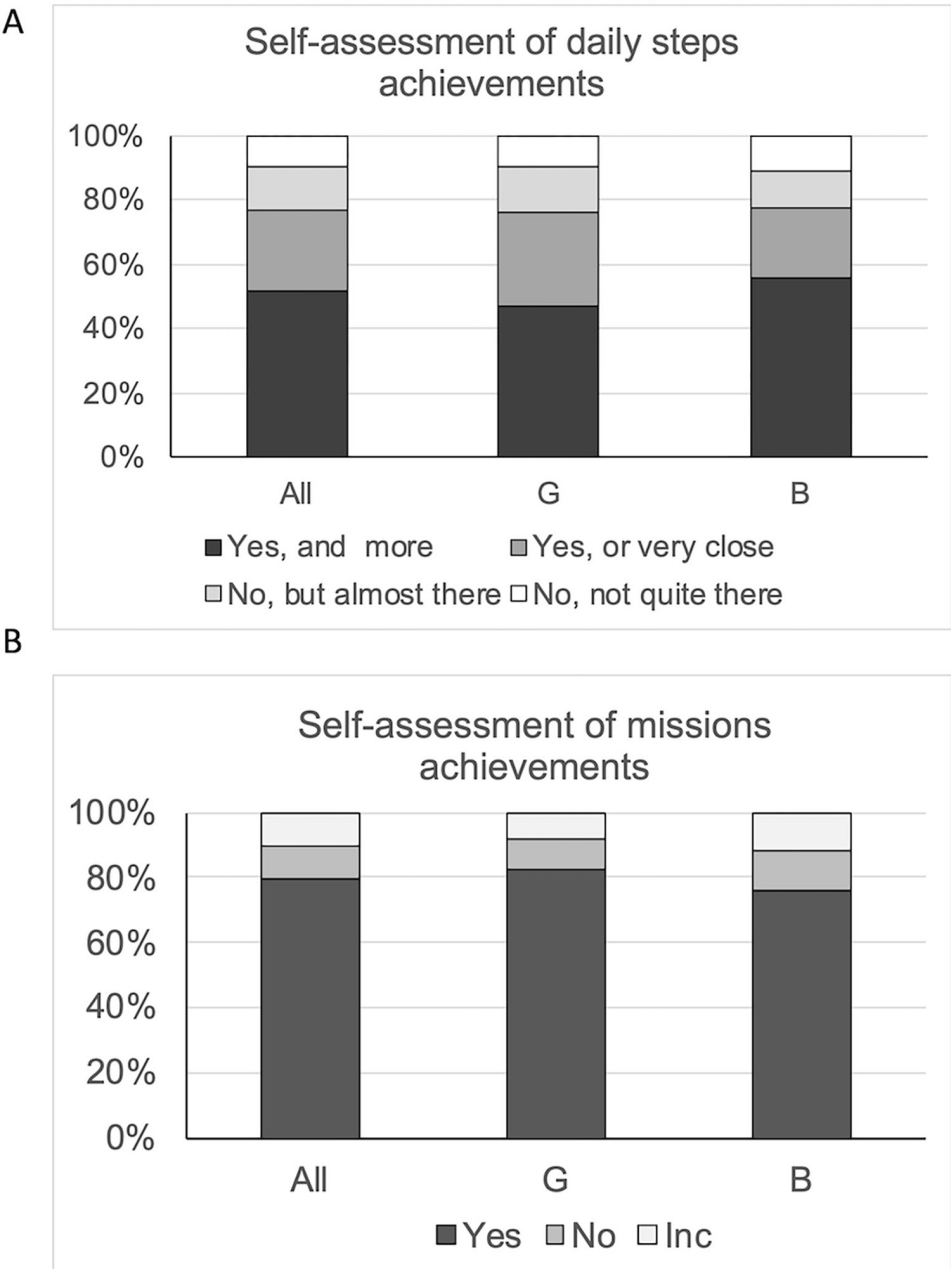
Self-assessment of achievements. Self-assessment of individual step goals achievements (A) and completion of missions (B) during the program (Inc: incomplete, no answer to the question). Results are expressed as percentage of participants. There was no significant difference between boys’ and girls’ report of achievements based on individual goals. All: all participants (n = 57), B: boys (n = 28), G: girls (n = 29).

Within each module, students entered the daily steps recorded by the activity tracker for each of the previous three days. Results showed that students met or surpassed goals in the 3 days preceding modules M2, M3, M4, M6, M9 and M10 while they missed out on achieving goals in M5 and M8 (Fig 4A). The proportion of days that the cohort achieved goals varied from 40% to 80% depending on the module as participants adjusted to step goals of increasing difficulty. Daily step goals were quite achievable at the beginning of the program and participants met or surpassed goals on most days (all: 72%, G: 70%, B: 79% of days following M2 and M3, Fig 4B). At the end of the program, while goals were much more challenging, participants met or surpassed their daily steps goals (i.e., their average daily steps met their goals) on 55% of the days (M9 and M10, Fig 4B). When asked to self-assess their achievements, participants said they had achieved or surpassed their daily step goals on 60% of the days in M2 and M3, and at least 50% of the days at the end of the program (M9, M10), showing a good interpretation of the step data they recorded in the app. Overall during the iEngage program participants said they either achieved or almost achieved their goals on 77% of days (with no difference between boys and girls G:76%, B: 77%; Fig 5A), while 10% across the program declared that they were “not quite there” and missed out. Overall, 79.5% of participants reported that they completed their mission (G:81%, B:78%, ranging from 62% to 89% across all modules, Fig 5B).

We assessed participants’ satisfaction with the program with a simple question at the end of each module ("did you have fun in completing the module") using a Likert scale (1 to 5). The average score for the whole program was 4.3 ± 0.99.

### Analysis of participants objective measures of physical activity using activity trackers data

**Fig 6:**
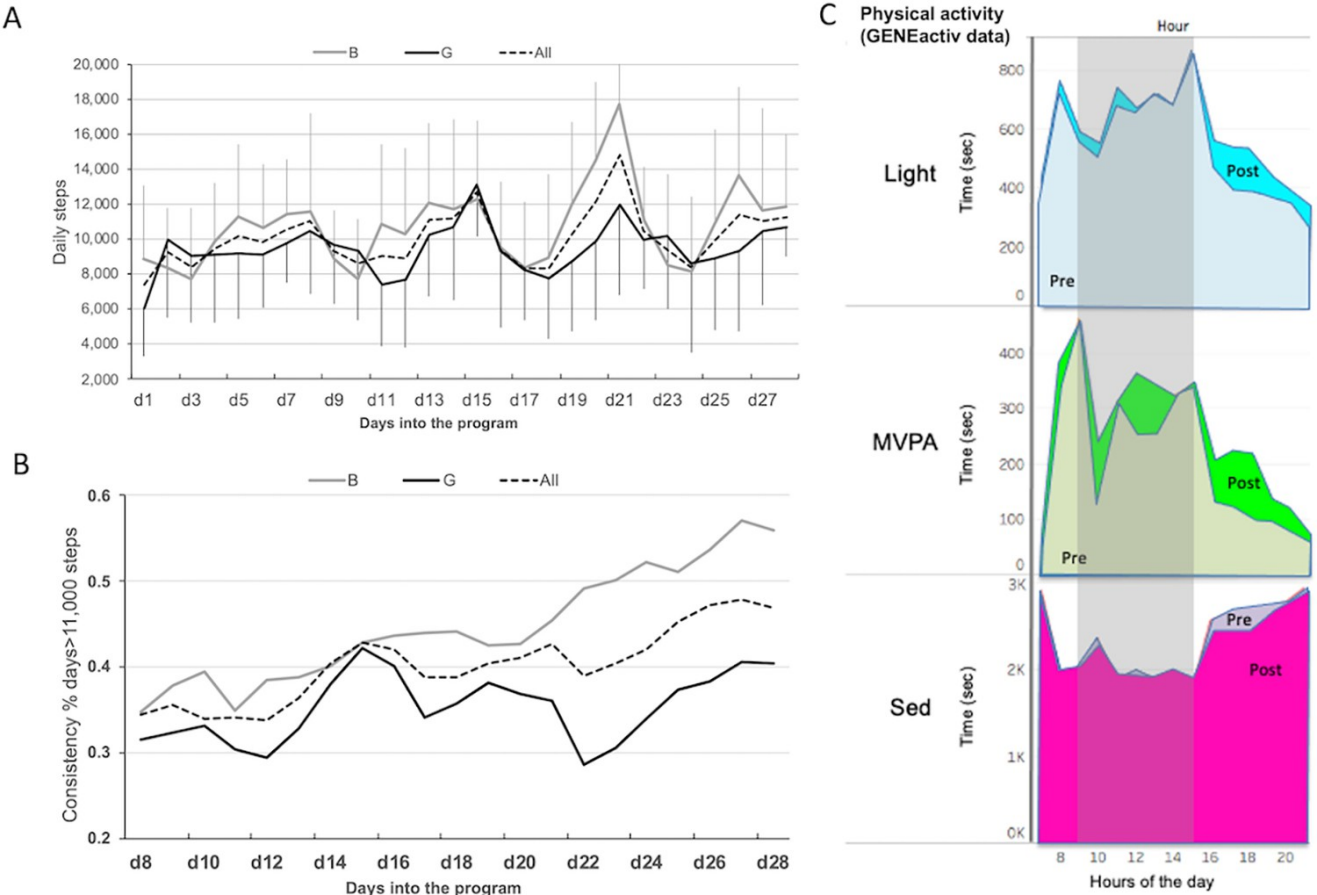
Objective PA data extracted from the Misfit trackers integrated in the platform or GENEactiv acidity trackers. Panel A: daily steps in all students, girls and boys, during the 5 weeks of the iEngage program. Panel B: consistency (%) of daily steps displaying at least 11,000 steps) over the course of the program. Panel C: hourly average of physical activity per intensity category (SED, light and MVPA) measure by GENEactiv trackers pre and post program from 8am to 8pm. This shows patterns of PA before and after the iEngage program during a school day. All: all participants (n = 57), B: boys (n = 28), G: girls (n = 29).

Objective continuous assessment of steps via Misfit Ray^©^ activity trackers during the program showed that both female and male participants increased their daily steps over time (Table 2, ANOVA with repeated measures all: F(1,55) = 12.2, *P* < .001). Comparing the first 4 with the last 4 days of program showed that daily steps increased by 2,647 steps/d (30% increase, all: 8,625 ± 1,263 versus 11,272 ± 1,471 steps/day, G: 8,545 ± 1,704 versus 10,155 ± 719 steps/day, B: 8,703 ± 895 versus 12,387 ± 1,070 steps/day; Table 2). During the program, daily steps dropped during weekends and raised again during school days with highest achievements seen after M5 and then M7 and M8 (Fig 6A).

**Table 2:** Physical activity assessed via physical activity questionnaire (PAQ), daily steps via wrist step tracker Misfit and moderate to vigorous physical activity (MVPA) assessed using GENEactiv wrist accelerometer.

Group		PAQ score	PAQ score	Daily steps	Daily steps	GENEactiv MVPA	GENEactiv MVPA
(G/B/All)		PRE	POST	First 4 days	Last 4 days	PRE	POST
						(min /day)	(min /day)
**Control**							
G	m	2.49	2.59			43.3	42.3
	SD	0.87	0.49			17	10
B	m	2.75	2.2			74.1^&^	61^&^
	SD	1.07	0.92			28	26.9
ALL	m	2.59	2.44			54.9	49.8
	SD	0.94	0.92			26.2	19.8
**iEngage**							
G	m	2.54	2.74	8545	10155 **	43.7	46.6
	SD	0.92	0.49	1704	918	17.6	21.6
B	m	3.02	3.08	8703	12387 **	57.2^&^	64.1^&^
	SD	0.87	0.66	994	1077	18.7	29.1
ALL	m	2.77	2.91 *^#^	8625	11272 **	49.6	54.3^#^
	SD	0.92	0.6	1263	1471	19.1	26.5

*: *P* = .04 Pre compared to post

^**#**^ Interaction effect group x time (PAQscore: *P* = 0.04; MVPA: *P* = .023)

**: *P* < .01 steps achieved at the end compared to the beginning of the program in each group

^**&**^: *P* < .01 gender effect in each group. Number of participants: control: n = 26 (PAQ) and n = 23 (GENEActiv data); iEngage: n = 57 (PAQ and Misfit sensor data) and n = 41 (GENEActiv data).

Consistency analysis showed that the proportion of days consistently displaying daily steps above 11,000 steps increased from 35% at the beginning to 48% at the end of the program, and up to 58% of days in male participants. Consistency was lower in girls compared to boys across the program with a more pronounced difference toward the end (Fig 6B). This difference between boys and girls is well aligned with the data reported in the platform by participants (Fig 4C). Objective data collected via GENEActiv accelerometers pre- and post- program or control indicated a significant increase in MVPA after iEngage and a significant difference between the iEngage and control groups (Table 2, 49.6 ± 19.1 pre- versus 54.3 ± 26.5 post- min/day in iEngage group; 54.9 ± 26.2 pre- versus 49.8 ± 19.8 post min/day in the control group, time*group, Willks’ Lambda, F(1,63) = 5.41, *P* = 0.023). Boys tended to increase MVPA more than girls (Table 1, time*group*gender, Willks’ Lambda, F(1,62) = 3.30, *P* = 0.073.

Analysis of physical activity patterns over the full school day showed that opportunities to spend more time in MVPA in response to the iEngage program were found during school time (recess and lunch breaks) and after school, about one hour after school until 7PM (Fig 6C). After school, both light and MVPA increased while time spent in sedentary time decreased.

## Discussion

This study is the first to report on the implementation and outcomes of a fully integrated mobile health program in primary school context, using both participant’s self-reported data and continuous monitoring of PA during the program. It shows that a digital health education and behaviour change program integrating activity tracker successfully guided adolescents to effectively progress PA goals and achievements. Data indicate that the program led adolescents to increase PA and MVPA in daily life through short bouts of activity during recess, lunch and after school. We show that the iEngage app can be used as a research platform to study how adolescents engage and respond to health education or behaviour change interventions.

### iEngage, goal settings and achievements

iEngage was based on BCTs, the Australian physical literacy framework and WHO recommendations for adolescents [16, 18, 29]. In addition, it encouraged connections with friends, siblings and parents through the accomplishment of missions, aiming to extend the program beyond the school context [12, 13]. While multicomponent school interventions address key determinants of PA, they are also complex, often resulting in poor implementation fidelity and low effectiveness [30]. Digital technologies provide the combined opportunity to enhance fidelity for program deployment, to record and evaluate process and to track engagement during the program [30]. iEngage takes the full advantage of digital technology through continuous recording of PA behaviour for 5 weeks and capturing all students’ interaction with the platform.

Recent systematic reviews and meta-analysis found interventions using diverse degree of mobile health technologies targeting the promotion of healthy behaviours in schools, however none used mobile health [31, 32]. None of the identified primary studies integrated activity trackers in an educational program and none were set to provide continuous recording and self-monitoring of PA during the intervention. While commercial PA apps exist and may provide an option, a study showed that only a few are suitable for children and adolescents [15]. In addition, these apps have a limited use of BCTs, provide poor information quality and do not take advantage of scientific evidence [15]. In addition, mobile apps alone or activity trackers alone do not seem to achieve change of health-related behaviour [22, 33]. Furthermore, studies showed that if students are not appropriately supported when provided with commercial activity trackers and apps, they may feel guilt and internal pressure particularly if a feeling of competition is developed or encouraged [22]. More recently new evidence-based digital apps designed for adolescents integrated functionalities including self-monitoring of health behaviours but did not integrate activity trackers, experiential learning and self-assessment [34]. By contrast, iEngage successfully promoted user engagement via several techniques and features including health and PA education, digital literacy uplift, self-paced goal settings, regular self-assessment of PA achievements against goals, an inclusive design, and missions that aimed to connect the program with the family as previously recommended [16]. These features and activities were supported through activity tracker data embedded in a structured framework using consistent wordings and graphics, allowing all participants to keep up with the process and to focus on learning and progressing their PA levels. iEngage promoted achievement of own goals rather than competing for highest PA levels. We found a good concordance between self-reported steps achievements and objective steps/day levels during the intervention, as well as sustained positive feedback indicating a high level of engagement with the program and the app [15]. This is important since self-efficacy, autonomy in goals-setting and self-assessment as well as intrinsic motivation, which are core to iEngage, predict the development of PA in young adolescents [35]. It is known that PA behaviour varies between school days and weekend days particularly in less active adolescents [36]. Continuous recording and analysis of daily steps over the duration of the program shows that daily PA behaviour did not change linearly and that continuous monitoring during intervention is key to understand patterns. Our study shows that the impact of the program on PA behaviour was stronger during school days compared to weekends, with sharp drop in daily steps over each weekend captured in the study. The effectiveness of a module delivered on Fridays was probably dampened by the change in environment and opportunities during the following weekend days. This suggests that future programs will need to include modules specifically targeting weekends with objectives and goals better adapted to family activities while aiming for maintaining a minimal acceptable level of PA.

### Impact of iEngage on MVPA

MVPA can only be assessed through research grade accelerometers which do not provide information to the user on their PA. This was done before and after the program in the iEngage group and before and after a 5-week period in the control group over the same weeks of the school term. At baseline, daily MVPA in all participants was on average ~50 min /day with higher MVPA engagement in boys (~63 min) compared to girls (~43 min), and only 32% of the cohort achieving 60-min/day MVPA on average [29]. Our data compare well with previous studies using activity trackers in similar age groups: average daily MVPA has been reported to be around 50 min, with more MVPA time in boys compared to girls in Australia [37, 38] or in the UK where the school system is comparable [36].

When analysing PA post- versus pre-intervention, previous systematic reviews and meta-analysis found no or little effect of school-based interventions, with no significant differences between those using or not web-based technology [30, 31]. Our study shows small changes, however, comparable to those obtained after much longer multicomponent school programs [30], indicating that our approach and a shorter program was effective. Analysis of hourly MVPA patterns indicated that 48% of the daily MVPA was achieved before the start of class, during lunch break, at recess and just after school (respectively contributing 15%, 10%, 11% and 11% of daily MVPA). On average school hours are more active and less sedentary than the rest of the day. Our results also showed that adolescents increased MVPA during lunch time and recess (+40% MVPA, +4 min) despite the relatively short duration of these periods, indicating that school time provides opportunities [29] for MVPA. Overall, our results demonstrate that, after completing iEngage, adolescents were able to effectively identify opportunities for MVPA, increasing MVPA levels in daily life via short bouts of activities. This may be a direct impact of the program because the missions specifically encouraged adolescents to identify periods during the day that offered opportunities for more activity, particularly with friends and siblings.

One study [37] found that MVPA was the most important activity behaviour for body composition in 11-12-years old and that conversion of sedentary time or light PA time was effective to prevent fat gain pointing to the importance of focusing on MVPA, rather than light PA or sedentary time, for intervention in children and adolescents. The authors found that in participants doing on average 23-min MVPA daily, the re-allocation of 30 min daily from sedentary time to MVPA was key to influence body composition. Our results, as well as those from others indicate that interventions with higher initial MVPA lead to an increase in MVPA in the range of 5 to 10 minutes. Shifting toward more MVPA, even in small amounts after school may have significant health impact since it has been shown that sedentary after school was often associated with recreational screen time and consumption of unhealthy foods [39]. Importantly, avoiding a decrease in MVPA is also an important target [37]. Our results indicate that participants in the control group decreased their MVPA during the same 5-week period (and same period of the calendar year), reinforcing the outcome of the iEngage program.

### Girls and boys respond differently to the program

Adolescent boys and girls differed from each other with regards to both PA levels and the way they responded to the program. While all students developed competences in setting goals, girls tended to be less ambitious with setting daily step goals during the whole program, progressing goals slightly slower than boys. This indicates that, although they were exposed to the same program, the pace at which girls were willing to progress was slower than for the boys. This attitude toward goals setting cannot be explained by physical capacity since girls in the program had similar or slightly better performance across all baseline fitness tests. However, we cannot exclude the impact of perceived fitness or social norms on goal setting [40]. While girls set goals at lower step levels during the program, they still struggled to achieve their goals after module 8 when the program encouraged participants to progress above 10,000 steps. Our results still show a significant progression of daily steps in girls, but a longer program and slower progression may be better suited to girls’ attitudes toward PA programs. Other studies suggested that girls may have less opportunities to further engage in PA afterschool [41], while differences between boys and girls with regards to intrinsic motivation or self-efficacy may also play a role [35].

While both girls and boys increased daily PA, boys were more successful than girls in increasing MVPA following iEngage. The greater enhancement of PA in boys compared to girls aligns with previous studies on school-based PA interventions including either single (PA) or combined (PA and nutrition) components [42] and with multicomponent school-based web-based health interventions [31]. More specifically, a comprehensive intervention tailored to 13-year-old adolescent girls, including sport, lunch time PA and seminars conducted over 12 months failed to increase PA assessed via accelerometry [43]. While it cannot be excluded that PA changed during the program, it was not possible to show any significant modification after the program [43]. A meta-analysis of interventions conducted in any setting in girls aged 12–18 years reported small size effects, large heterogeneity and concluded that behaviour change in girl adolescents will likely be challenging but that school intervention may be more effective [41].

### Limitations

One limitation of this study is the relatively small cohort however our results were consistent across the two schools. In each school, all year 5 and 6 students were included indicating that a whole class can participate in the program. iEngage is a short program with 10 modules delivered over 5 weeks. While this allows for strong focus and facilitates sustained motivation, a slower pace may be envisaged to enable progression and maintenance at slower rates, which may be more suitable for female adolescents. This study specifically focused on PA changes during the program and immediately after, however allowing the adolescents to keep the Misfit Ray^©^ activity tracker and to synchronise weekly with the app once the program is completed could encourage sustaining the behaviour over longer periods.

### Conclusions and further directions

Delivered through a tailored digital environment, iEngage combines health education, experiential learning, interaction with peers and behavioural change theory. It enables individual goal setting, self-monitoring and self-assessment. In addition, iEngage provides researchers with a powerful tool to study how adolescents respond to information, activities and goals setting through continuous recording embedded in the app. In the future, iEngage could be considered to help adolescents maintain or increase PA in situations such as pandemic-induced lockdown and remote school activities but also in paediatric populations in which PA behaviour is important.
